# Mediating effect of empowerment on the relationship between global function and personal recovery among community-dwelling patients with schizophrenia: a cross-sectional study

**DOI:** 10.1186/s12888-021-03239-6

**Published:** 2021-05-07

**Authors:** Kuen Tai Lee, Shih Kai Lee, Mei Jou Lu, Wen Ling Hsieh, Wen I. Liu

**Affiliations:** 1 National Taipei University of Nursing and Health Sciences, Taipei, Taiwan; 2 Department of Nursing, Tsaotun Psychiatric Center, Ministry of Health and Welfare, Nan-Tou, Taiwan; 3 Shu-Zen Junior College of Medicine and Management, Kaohsiung City, Taiwan

**Keywords:** Global function, Empowerment, Personal recovery, Mediating effect, Community-dwelling patients with schizophrenia

## Abstract

**Background:**

Functional degradation among community-dwelling patients with schizophrenia can negatively influence their recovery. Given the importance of patient empowerment during recovery, this study examined the mediating effect of empowerment on the relationship between global function and personal recovery among community-dwelling patients with schizophrenia.

**Methods:**

This cross-sectional study recruited community-dwelling patients with schizophrenia from northern and central Taiwan. Questionnaires with verified reliability and validity were provided and collected on site by trained nurses. Global function, empowerment, and personal recovery were measured using the Global Assessment of Functioning (developed by the American Psychiatric Association), Empowerment Scale, and Questionnaire on the Process of Recovery, respectively. The causal steps approach proposed by Baron and Kenny and the Sobel test were utilized to verify the mediation effect. The causal steps approach tested the four following pathways (regression coefficients): global function on empowerment (Path a), global function and empowerment as predictors of personal recovery (Path b), global function on personal recovery (Path c), and global function and empowerment on personal recovery (Path c’).

**Results:**

A total of 373 participants completed the survey. After controlling for factors associated with recovery, Paths a (β = .24, *p* < .001), b (β = .68, *p* < .001), and c (β = .19, *p* < .001) were found to be significant; however, Path c’ was not significant (β = .03, *p* = .452). Empowerment was determined to exert “full mediation” over the effects of global function on personal recovery, and the Sobel test indicating significant mediation (Z = 3.61, *p* < .001).

**Conclusions:**

Empowerment fully mediates the association between global function and personal recovery. This study suggested that offering empowerment-oriented care services may be more effective than global function improvement in recovery among these patients.

## Background

Schizophrenia is a complex chronic mental illness characterized by delusions, hallucination, or confusion in speech and behavior. Cognitive impairment has been shown to affect global functions, leading to employment difficulties and social withdrawal, which consequently influence their recovery [1–3]. Approximately 152,110 individuals in Taiwan have schizophrenia, with medical expenses as high as 11.2 billion Taiwan dollars [4]. A study that tracked 200 schizophrenia cases for 20 years found that 57% of patients often have delusional symptoms that interfere with recovery [5]. Despite continuous treatment, 23.7% of the cases developed negative symptoms [6], while 75% exhibited poor functional outcomes [7], which affect the degree of recovery and necessitate long-term continuous care [8–10].

Recovery from schizophrenia can be segmented into clinical and personal recovery. Neurobiology emphasizes clinical recovery, which is the restoration of symptomology, neurocognition, and objective social function [11]. Patients with psychiatric problems define personal recovery as the attainment of a meaningful and valued life rather than the simple absence of symptoms. Personal recovery can also be a care outcome [12–14]. Scholars proposed the CHIME framework for personal recovery, with elements including connectedness, hope, and optimism about the future, identity, meaning of life, and empowerment [15]. Despite suffering from a chronic mental illness, when patients focus on their current lives and autonomy, they can still have hope and live a worthwhile life and contribute to society, rather than merely waiting for a cure [11].

Notably, global mental health care aims to improve recovery, with developed countries already having successfully developed recovery programs and effectiveness evaluations [12, 16, 17]. Moreover, mental health services must be human-, community-, and recovery-oriented [18] considering that the extent of personal recovery can impact the quality of life of the community-dwelling patients with mental illness [19, 20]. However, follow-up studies and meta-analyses have reported that recovery rates among individuals suffering from mental illness only ranged from 13.5 to 37.9% [14, 21, 22]. Furthermore, patient recovery from schizophrenia will mitigate health costs [23]. Hence, promoting recovery among patients with mental illness is urgently needed.

Several systematic reviews and follow-up studies have identified the following influence factors for recovery in patients with schizophrenia: gender, age, employment status, age of onset, illness duration, psychiatric symptoms, global function, side effects, therapeutic alliance, insight, and medication adherence [9, 13, 22, 24, 25]. Among such factors, global function has been identified as an important factor of recovery among patients with schizophrenia [9, 25, 26]. However, some systematic reviews have revealed that rehabilitation or psychoeducation programs developed to improve global function and achieve better recovery for individuals with schizophrenia were ineffective [15, 27]. Schizophrenia is a debilitating chronic disease that impacts all major life areas. Given that substantial improvements may become difficult as the disease progresses, the empowerment concept has emerged as a novel approach to recovery-oriented interventions for community-dwelling patients with schizophrenia.

Empowerment plays a critical role in the recovery of patients with mental illness [9, 15, 26, 28, 29]. A systematic review that included 97 articles and redefined the recovery model, called the CHIME model, identified empowerment as one of the elements for recovery [15]. Empowerment mainly entails promoting patient autonomy, independent decision-making and responsibility, and self-management [30, 31]. Additionally, empowerment can mediate the association between the function and quality of life in patients with schizophrenia [32–34], the association between the in-group perceptions and personal recovery among people with mental illness [32], and the association between rehabilitation activities and quality of community-based life for people with schizophrenia [33].

In summary, even after prolonged functional rehabilitation, patients with schizophrenia find it difficult to improve global functions, and unfortunately, global function influences recovery [15, 27]. Although studies have suggested that empowerment is an element of recovery [9, 32, 35], few have explored the mediating effect of empowerment on the global function needed for recovery. Understanding whether empowerment mediates the association between global functioning and recovery could serve as a novel treatment target for promoting personal recovery. Therefore, this study examined the mediating effect of empowerment on the relationship between global function and personal recovery among community-dwelling patients with schizophrenia. Based on the current literature, we hypothesized that empowerment mediates the association between global function and personal recovery in patients with schizophrenia.

## Methods

### Study design, participants, and procedures

This cross-sectional study utilized convenience sampling. This study had been reviewed and approved by the Human Test Review Committee of the community psychiatric department of two psychiatric hospitals in Taiwan before data collection from September 1, 2016 to April 30, 2017.

Participants were community-dwelling patients with schizophrenia who received community care services, such as outpatient, daycare, and home treatment. Those who satisfied the DSM-5 criteria for schizophrenia, were able to communicate in Mandarin and Taiwanese, did not indulge in substance abuse, and did not reside at mental rehabilitation institutions were included as research subjects.

The researcher first explained the purpose of the research and asked the participants to fill out the questionnaire by themselves after obtaining consent. Data were only collected once per participant, with data collection lasting approximately 30 min. Those who wished to terminate participation midway through the study were allowed to do so to avoid answering deviations related to forced participation.

### Measures

The basic data sheet includes the participants’ general and illness information, including gender, age, education, employment, marital status, age of onset, duration of illness, and number of hospitalizations. The remaining variables were measured using the following structured questionnaires with good reliability and validity.

#### Global assessment of functioning (GAF)

GAF is an assessment tool proposed by the American Psychiatric Association. The single-term GAF uses a 0–100 Likert scale to measure global function, with higher scores indicating better global function. The infraclass correlation coefficient of GAF was 0.89–0.95 and showed good reliability, with higher scores indicating better function [33, 36].

#### Empowerment scale

The empowerment scale is a self-filled scale developed by Rogers et al. in 1997 [37] that uses a Likert scale, with 1 point indicating strong agreement and 4 points indicating strong disagreement, over a total of 25 questions. After reverse scoring the questions, higher scores indicated higher empowerment.

Taiwanese scholars have translated and verified the reliability and validity of this scale in patients with mental illnesses and have revised it into a 13-question Chinese version. The total score ranges from 13 to 52 points. It covered three factors, namely, self-efficacy, community action, and emotional control [38].

#### Questionnaire on process of recovery (QPR)

The QPR is a self-filled scale developed by Neil et al. in 2009 [39] that uses a Likert scale ranging from 0 to 4, with a total of 22 questions. The total score can range from 0 to 88, with higher scores indicating better personal recovery. The scale covered three factors, namely, self-empowerment, effective interpersonal relationships, and life reconstruction, with the Chinese version of the QPR having a Cronbach’s α of .90 [40].

#### Brief psychiatric rating scale (BPRS)

The 16-item BPRS was developed by Overall and Gorham in 1962 [41] to measure psychiatric symptoms, and it has good construct validity and retest reliability (r = .78, *p* < .001) [42]. The Chinese version of BPRS is scored from 0 to 6 points, with the total score ranging from 0 to 96 points. Higher scores indicate more psychiatric symptoms [43].

#### Medication adherence rating scale (MARS)

MARS is a 10-question self-administered questionnaire developed by Thompson et al. [44] that is scored between 0 and 10 points, with higher scores indicating better medication adherence. Kao and Liu had translated this scale into Chinese, with a Cronbach’s α value of .72, retest reliability after 2 weeks of .80 (*p* < .01) [45].

#### Glasgow antipsychotic side-effect scale (GASS)

GASS is a 22-item questionnaire developed by Waddell and Taylor in 2008 [46] that uses a Likert scale ranging from 0 to 3 points, with a total score of 0–63 points. Higher scores indicate more serious drug side effects. This scale had a Cronbach’s α of .79 [47], whereas its construct validity was associated with LUNSERS [46].

#### Working Alliance inventory-short (WAI-S)

The 12-item WAI-S uses a 7-point Likert scale to measure therapeutic alliance, with higher scores indicating better therapeutic alliance [48]. WAI-S had a Cronbach’s α of .90, with good reliability and validity [49].

#### Schedule for assessment insight in psychosis (SIP)

The SIP is a 9-item questionnaire developed by Yen et al. [50] that uses a Likert scale ranging from 1 to 4 points, with a total score of 9–36 points. Higher scores indicate better insight. This scale had a Cronbach’s α of .92, which was related to the Scale to Assess Unawareness of Mental Disorder and Schedule for the Assessment for Insight, with good reliability and validity [50].

### Statistical analyses

To estimate the sample size using G power 3.1.9.4 [51], we set the F test to “linear multiple regression: fixed model, R2 deviation from zero” and R2 to .35 based on a previous study [33]. Thereafter, we calculated the effect size f2 to be 0.54, with α at .05, β at .80, and the number of predictors at 15. Ultimately, at least 150 samples were required, and 373 were needed for a sufficient statistical power.

SPSS software package (New York: IBM) was used for data analysis. Frequencies, percentages, averages, and standard deviations were used to describe the distribution of each variable. Considering the different score ranges of the original scale, we converted the scores into percentage to better understand the degree of each variable (mean score/total score of the scale * 100%). The independent-samples t-test, ANOVA, and Pearson correlation were used to analyze the relationship between the independent variable and recovery.

After identifying the factors related to recovery, we included them in the control variables of the mediation model. Global function was defined as the independent variable (X), empowerment as the mediating variable (mediator; M), and recovery as the dependent variable (Y). Baron and Kenny’s path analysis and Sobel test were adopted to verify the mediating effect of empowerment [52]. Baron and Kenny’s path analysis uses regression to test the following four paths: (1) Path c: X on Y; (2) Path a: X on M; and (3) Path b: X and M as predictors of Y; if these three pathways were found to be significant, the mediating effect was said to be established. (4) Path c’: X and M on Y. A significant Path c’ indicated partial mediation, whereas nonsignificant Path c’ indicated full mediation. To double check whether the mediation effect exists, we used the Sobel test, which is a very conservative method for preventing negative variance estimate. If the Sobel test z-score is greater than 1.96, the mediation effect is interpreted to be statistically significant [53].

## Results

### Demographic characteristics and relationships with recovery

A total of 373 community-dwelling patients with schizophrenia (average age = 46.61 years; 58.2% males) participated herein. Details regarding their demographic characteristics are presented in Table 1.

**Table 1:** Participant characteristics and relationships with recovery (*N* = 373)

Variables	Data distribution		Relationship with recovery (QPR)		
	N (%)	Mean (SD)	Mean (SD)	*t*^a^/*r*^b^/*F*^c^	*p*
Sex				−.11^a^	.915
Male	217 (58.2)		60.72 (13.35)		
Female	156 (41.8)		60.87 (12.50)		
Age		46.61 (9.10)		.04^b^	.412
Education level				1.36^c^	.257
Middle school or below	137 (36.7)		59.33 (11.65)		
Senior high school	161 (43.2)		61.55 (12.19)		
College and above	75 (20.1)		61.77 (16.43)		
Marital status				1.20^c^	.304
Single	264 (70.8)		61.36 (13.32)		
Married	58 (15.5)		60.24 (10.25)		
Divorced/widower	51 (13.7)		58.37 (13.86)		
Employment				−2.45^a^	.015
No	285 (76.4)		59.87 (13.37)		
Yes	88 (23.6)		63.73 (11.19)		

Note: a, *t*-test; b, Pearson’s correlation (*r*); c, ANOVA (*F*); Questionnaire on Process of Recovery (QPR)

Among the participant characteristics, a significant relationship was observed between employment status and recovery (t = − 2.45, *p* = .015), whereas gender, age, education level, and marital status showed no significant relationship with recovery.

### Distribution of disease factors, therapeutic factors, global function, and empowerment and their associations with recovery

The mean empowerment score was 34.80 (converted score percentage = 55.9%), whereas the mean recovery score was 60.78 (69.1%) (Table 2).

**Table 2:** Distribution of related variables and their associations with recovery (*N* = 373)

Variables	Data distribution		Relationship with recovery	
	M (SD)	Score percentage	Pearson's correlation (*r*)	*p*
Age of onset	24.20 (7.29)	–	.01	.856
Illness duration	22.42 (9.15)	–	.04	.474
Psychiatric symptoms	12.29 (7.25)	12.8%	−.31	<.001
Number of hospitalizations	5.44 (6.20)	–	.03	.628
Global function	69.5 (15.28)	69.5%	.23	<.001
Side effects	11.99 (10.38)	19.0%	−.22	<.001
Therapeutic alliance	60.11 (14.24)	66.8%	.39	<.001
Insight	25.02 (5.52)	59.3%	.27	<.001
Medication adherence	6.04 (2.44)	60.4%	.37	<.001
Empowerment	34.80 (4.50)	55.9%	.77	<.001
Recovery	60.78 (12.98)	69.1%	–	–

Among the main variables, psychiatric symptoms (*r* = −.31, *p* < .001), global function (*r* = .23, *p* < .001), drug side effects (*r* = −.22, *p* < .001), therapeutic alliance (*r* = .39, *p* < .001), insight (*r* = .27, *p* < .001), medication adherence (*r* = .37, *p* < .001), and empowerment (*r* = .77, *p* < .001) had a significant relationship with recovery. Variables not significantly related to recovery included age of onset, illness duration, and number of hospitalizations (Table 2).

### Mediating effects of empowerment on the relationship between global function and recovery

**Fig. 1:**
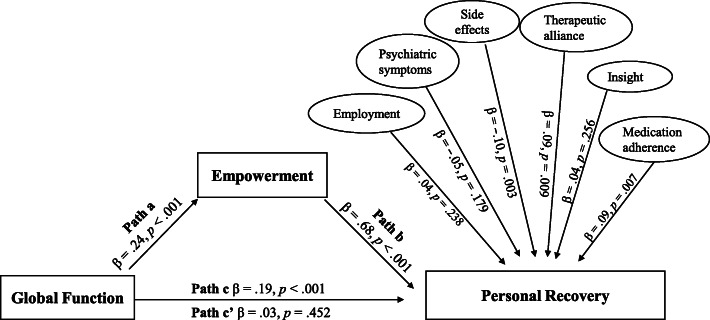
Mediating effects of empowerment on the relationship between global function and personal recovery

This study initially controlled for variables significantly related to recovery (employment, psychiatric symptoms, side effects, therapeutic alliances, insight, and medication adherence) to test the mediation model, for which the variance inflation factor (VIF) ranged from 1.14 to 1.55, and the values of VIF were not greater than the critical index 10, which indicated that there was no problem of multiple collinearity among the independent variables. And subsequently verified Path a (β = .24, t = 4.32, *p* < .001), Path b (β = .68, t = 19.84, *p* < .001), and Path c (β = .19, t = 3.65, *p* < .001). Accordingly, all three paths were significant. When considering global function and empowerment simultaneously, a nonsignificant regression coefficient was found for Path c’ (β = .03, t = .75, *p* = .452), suggesting that empowerment was a full mediation between global function and personal recovery. After establishing the model, the Sobel test also found a significant mediation effect (Z = 3.61, *p* < .001) (Fig. 1).

## Discussion

This study included a large sample of community-dwelling patients with schizophrenia to examine the mediating effect of empowerment on the relationship between global function and recovery. Results revealed that the empowerment and recovery levels were 55.9 and 69.1%, respectively, which are slightly higher than those presented in other studies [14, 22]. This aspect may have been related to participants who have received various community care services, such as outpatient clinics, day wards, or home treatment services. Therefore, community care is important for promoting recovery from mental disorders in community settings.

Our results identified employment, psychiatric symptoms, global function, side effects, therapeutic alliances, insight, medication adherence, and empowerment as recovery-associated factors. These results may be related to recovery factors such as relationships with others, self-identity and hope, and participation; for example, if they can establish connections with the community and others at work, show their social role and self-worth, and promote personal recovery [29]. Therapeutic relationships can help establish interpersonal connections [54], and insight is related to disease acceptance, which can help patients recover [29]. However, based on the mediation model of this study, the most predictable variables for recovery are empowerment, medication adherence, and therapeutic alliance, consistent with the connotation of CHIME and the results of systematic literature [15, 29].

The results of this study indicate that global function predicts empowerment, which then predicts personal recovery. Empowerment was determined as a mediator, which is consistent with the findings of previous studies [32–34]. Our study shows that empowerment mediates the relationship between global function and personal recovery, and we can target empowerment in an attempt to reduce the impact global function has on personal recovery.

In actual situations where global function is difficult to modify immediately, our research has verified that empowerment can fully mediate the influence of global function on recovery. Despite the relationship between global function and degree of empowerment, limited general cognitive function can affect the degree of empowerment given its requirement of self-reflection, which consequently affects recovery owing to the close relationship between degree of empowerment and recovery [28, 31, 35, 55]. This study verified that empowerment is indeed an important element for recovery, despite the negative impact of global function on recovery [9, 32]. Traditionally, mental health practices had emphasized strategies for enhancing the global function of patients. However, we believe that shifting toward relevant strategies for empowering patients is necessary and may be more effective for patient recovery than enhancing global functions.

In the clinical setting, the results presented here provide professionals with an alternative approach to care. Transitioning to an empowerment-oriented care strategy can effectively reduce the negative impact of poor global function on recovery. Empowerment-oriented care addresses the establishing of partnerships, focusing on personal strengths and sharing in the responsibility of decision-making, inspiring a sense of hope and generating motivation, enhancing patients’ self-management ability, and linking up support networks, including self-maintenance during illness and life and familial–societal connections [30, 56, 57]. Empowered care also includes reducing stigma, formulating required care according to individual needs, and helping them increase social participation [56].

### Limitations

To our knowledge, this has been the first study to focus on the mediating effect of empowerment on the relationship between global function and recovery in schizophrenia after accounting for multiple related factors. Nonetheless, some research limitations of the study are worth noting. First, given the cross-sectional, descriptive design that lacked causal inference, our results should be interpreted with caution. Second, the main dependent recovery variable was the self-evaluated recovery scale, which may differ from the actual recovery status that contains specific indicators. Third, although the constructs of empowerment and personal recovery partially overlap, this study confirmed that these variables have no collinearity. We suggest that future studies use measures that have a lower degree of overlap. Future longitudinal studies are thus needed to identify variables at different time points, thereby deepening our knowledge regarding the mediating effect of empowerment on the relationship between global function and recovery.

## Conclusions

Empowerment was found to exert full mediation over the effects of global function on personal recovery. This study suggests that developing and applying empowerment-oriented community care may be more effective in promoting psychiatric recovery than focusing on global function among community-dwelling patients with schizophrenia. Despite attempting to include all active variables affecting recovery, some may have still been missed. Future investigations on the relationships between empowerment and recovery might include additional variables. Developing empowerment- and recovery-oriented services for community-dwelling patients with schizophrenia and then utilizing carefully designed randomized controlled trials to investigate the effectiveness of such programs on improving psychiatric recovery are also essential.

## Data Availability

Datasets used and analyzed herein are available from the corresponding author upon reasonable request.

## Abbreviations

BPRS: Brief Psychiatric Rating Scale
GASS: Glasgow Antipsychotic Side-effect Scale
M: Mediator
MARS: Medication Adherence Rating Scale
QPR: Questionnaire on Process of Recovery
SIP: The Schedule for assessment Insight in Psychosis
WAI-S: Working Alliance Inventory-Short
